# Supplementary material for: Neuroprotective Effect of Dioscin against Parkinson’s Disease via Adjusting Dual-Specificity Phosphatase 6 (DUSP6)-Mediated Oxidative Stress
Source: Molecules. 2022 May 14;27(10):3151. doi: 10.3390/molecules27103151 (PMC9146847; doi:10.3390/molecules27103151)
Supplement: Supplementary file 1 [file molecules-27-03151-s001.zip › molecules-1699692-supplementary.pdf]

Supplementary Materials

Table S1. Primary antibodies.

Antibody	Source	Dilution	Company	Cat. no.
DUSP6	Rabbit	1:1,000	Zen Bio, Chengdu, China.	381008
P-ERK	Rabbit	1:1,000	ProteinTech Group, Inc.	28733-1-AP
ERK	Rabbit	1:1,000	ProteinTech Group, Inc.	16443-1-AP
Keap1	Rabbit	1:1,000	ProteinTech Group, Inc.	10503-2-AP
Nrf2	Rabbit	1:1,000	ProteinTech Group, Inc.	16396-1-AP
HO-1	Rabbit	1:1,000	ProteinTech Group, Inc.	27282-1-AP
SOD	Rabbit	1:1,000	Zen Bio, Chengdu, China.	350163
GAPDH	Rabbit	1:5,000	ProteinTech Group, Inc.	10494-1-AP
